# A new approach to classifying polymer type of microplastics based on Faster-RCNN-FPN and spectroscopic imagery under ultraviolet light

**DOI:** 10.1038/s41598-024-53251-5

**Published:** 2024-02-12

**Authors:** Thunchanok Thammasanya, Sakarat Patiam, Eknarin Rodcharoen, Ponlachart Chotikarn

**Affiliations:** 1 Faculty of Environmental Management, Prince of Songkla University, Hat Yai, Thailand; 2 Coastal Oceanography and Climate Change Research Center, Prince of Songkla University, Hat Yai, Thailand; 3 Aquatic Science and Innovative Management Division, Faculty of Natural Resources, DoE for Sustainable Aquaculture, Prince of Songkla University, Hat Yai, Thailand

**Keywords:** Environmental sciences, Ocean sciences, Mathematics and computing

## Abstract

Hazardous compounds from microplastics in coastal and marine environments are adsorbed by live organisms, affecting human and marine life. It takes time, money and effort to study the distribution and type of microplastics in the environment, using appropriate expensive equipment in a laboratory. However, deep learning can assist in identifying and quantifying microplastics from an image. This paper presents a novel microplastic classification method that combines the benefits of UV light with deep learning. The Faster-RCNN model with a ResNet-50-FPN backbone was implemented to detect and identify microplastics. Microplastic images from the field taken under UV light were used to train and validate the model. This classification model achieved a high precision of 85.5–87.8%, and the mAP scores were 33.9% on an internal test set and 35.7% on an external test set. This classification approach provides a high-accuracy, low-cost, and time-effective automated identification and counting of microplastics.

## Introduction

Today, plastics are the most adaptable and widely used materials. Plastic production on a global scale reached nearly 368 million tons in 2019 and is expected to reach 1.1 billion tons by 2050^1^. Unscrupulous waste management has led to the release of plastic waste into the environment. In the marine ecosystem, the amount of plastic that enters the ocean each year is between 4 and 12 million metric tons, and by 2040, that number will rise to 29 million metric tons^2^. This marine debris affects a variety of marine species. Numerous animals such as fish, sea turtles, and many more, suffer and die after eating plastic or after being stuck in it^3–5^. Moreover, instead of entirely degrading, plastics become shredded and broken down into fibres and tiny fragments, and those of size less than 5 mm are known as microplastics^6–8^. Microplastics are also found in products like toothpaste and face cleansers. They are abundant in coastal and marine environments and contain and adsorb hazardous chemicals^3,9^. Humans may be exposed to chemical contaminants through the consumption of organisms that have consumed contaminated microplastics, possibly with biomagnification of the contaminants via trophic transfer^10,11^. Even though there is no evidence of the effects of microplastics on humans in a broad population, in a lab setting the microplastics can harm human cells, causing both allergic reactions and cell death^4^. According to some reports, microplastics can contribute to respiratory problems and colorectal cancer^5,12^. In addition, numerous studies on marine life have shown that fish that consume microplastics have early mortality, energy depletion, reproductive problems, behavioural issues, and gut obstructions. The impact of these microplastics also extends to marine ecosystems, such as seagrass meadows, mangroves, and coral reefs^5,13^.

Microplastics are a globally recognised and growing environmental concern. Precise measurements of the amount of microplastics in the environment and the identification of microplastic types are needed in order to understand and evaluate the complexity of the problem and to choose the top mitigation priorities. Moreover, for the purpose of monitoring microplastics, reliable and comparable sampling and analytical procedures are essential. Extraction, separation, identification, and quantification are the steps in the analytical process applied to microplastics in environmental samples. Several techniques for microplastic identification are available. The simplest method for the identification of microplastics is visual identification. The stereo microscope is the most frequently used identification tool in microplastic studies, for counting and sorting microplastic particles according to colour, size, brightness, and morphology^14–16^. However, challenges remain since the previous studies are unable to create a uniform classification of microplastics data for the various microplastics in nature, covering their different forms, colours, and polymer types. So, visual sorting should be combined with chemical composition analysis to better identify the polymer type. Currently, pyrolysis or thermal decomposition gas chromatography coupled with mass spectrometry, Fourier transform infrared (FTIR) spectroscopy, or Raman spectroscopy, are the most prevalent techniques for identifying microplastics chemically. However, the visual identification method takes more time and can miss certain tiny, translucent particles that are difficult to recognize. Although FTIR and Raman mapping might mitigate this problem, access to pricey analytical equipment might not be feasible^12^; novel methods should be developed. Physical (size, shape, and colour) and chemical (polymer type) aspects are the two crucial parameter types in microplastic analysis^12^. Because determining key features like particle type or size involves time-consuming and intensive manual labour, counting and classifying certain types of particles is preferably automated using image analysis based identification methods. Using neural networks with computer image processing to overcome limitations may be a viable option. The methods currently used in image-related research that are speedy, reliable, repeatable, and highly efficient are those based on computer vision and deep learning.

Several studies have employed machine learning and computer vision in analysis to quantify and categorize microplastics^17–20^. The results have indicated a highly accurate classification. However, microplastics used in previous studies are not necessarily similar to those collected in the field, and their image analysis necessitates the use of specialized equipment, such as special staining dyes and illumination^12,21^, a high-resolution scanner^19^ or microscopy images^20^. Hence, it is important to develop protocols that are both affordable and effective in detecting microplastics. The methodology proposed in this work is based on images captured by a camera under UV light, which is a low-cost approach that has been developed to create inexpensively photos showing fluorescent response of microplastics^22,23^. Furthermore, introducing deep learning techniques improves the performance of computer vision.

The purpose of this study was to demonstrate an innovative automated, low-cost, and reliable method using artificial intelligence, that is capable of detecting and classifying microplastics. Additionally, this study demonstrates how addressing certain knowledge gaps may aid in further improving a standardized protocol for microplastics quantification and identification.

## Methodology

A high-level overview of the classification process, applied to the samples collected, is now provided. First, microplastic images were acquired under ultraviolet light. Second, the microplastic images were well-annotated and implemented as a dataset. Third, the data were enhanced and more data was generated to increase the number of images. The dataset was then fed into the Faster-RCNN model training. Finally, this model was used for quantifying and classifying microplastics. The following subsections contain additional information about each of these stages.

### Study area

**Figure 1:**
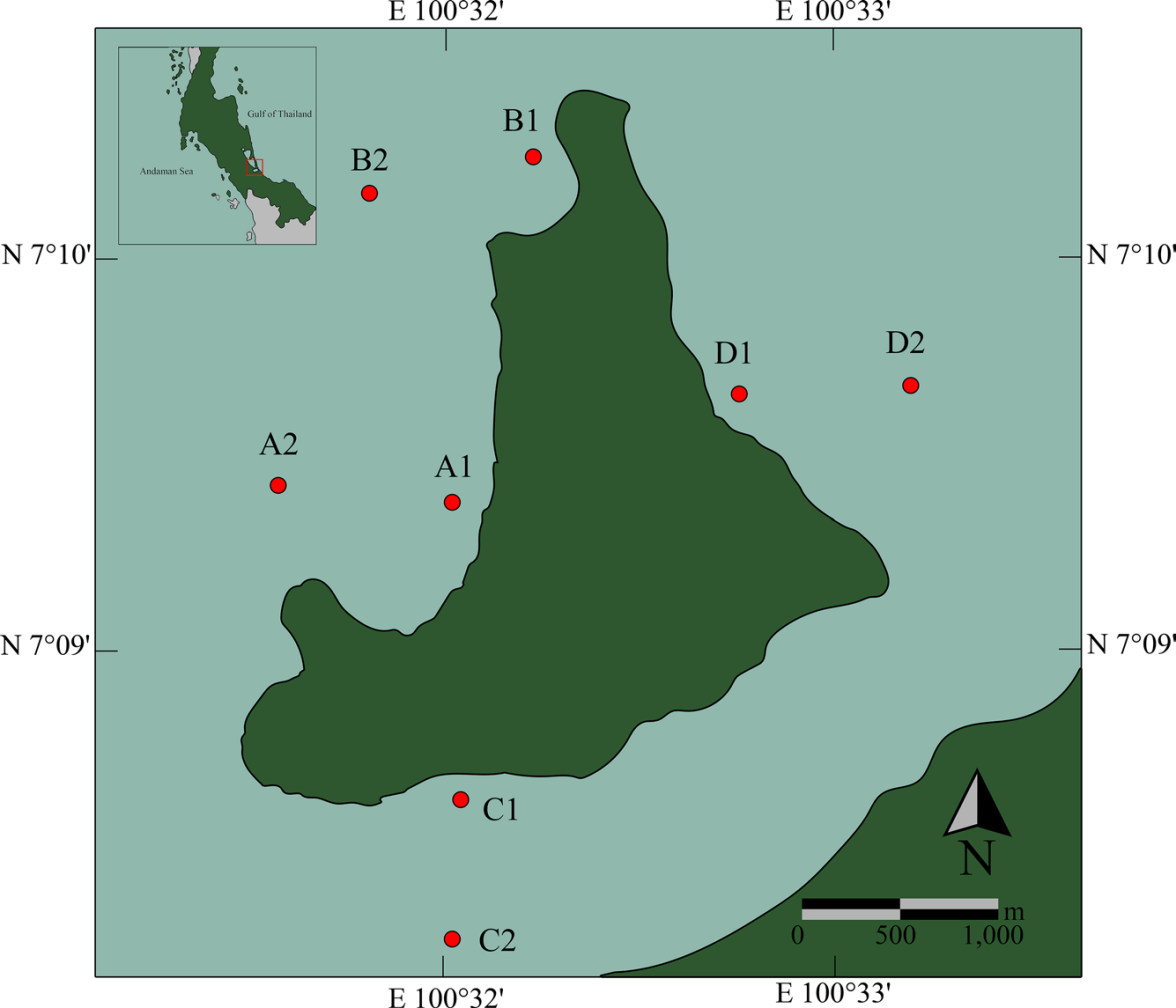
The study areas around Koh Yo island in Songkhla lagoon. The map was created using Google Earth Pro (Version 7.3.6.9750) and post-processed with Adobe Photoshop (Version 25.3.1).

Microplastic samples were collected from nearshore and offshore around Koh Yo in Songkhla province, Thailand, between May 2019 and February 2020 (Fig. 1). Station A was in Wat Thai Yo, which practices community-based aquaculture (7°9′45.10″ N, 100°32′10.46″ E), Station B was in Ban Ao Sai area (7°10′48.64″ N, 100°32′25.55″ E), having many structures including households, homestay, restaurants, and fish cages in the area. Station C was located in a mangrove area (7°8′51.27″ N, 100°32′9.01″ E), and station D was on the eastern side of Koh Yo (7°10′3.50″ N, 100°33′0.08″ E), which is an island in the Songkhla lagoon.

### Sample collection, image acquisition and identification

Samples were collected from 8 stations (4 stations along the shoreline and 4 stations offshore) around Koh Yo island between May 2019 and February 2020. Three repeat samples of 100 L of water were collected using a plankton net with a 50 µm mesh size. Three repeat samples of soil and benthic fauna were collected from the lagoon bottom using a 15 × 15 cm Ekman grab sediment sampling tool^6,24,25^. A study of the microplastics accumulating in sediment used a saturated sodium chloride solution (NaCl)^6,26^ and H_2_O_2_ for benthic fauna^27^. The microplastics were cleaned with a 30% solution of hydrogen peroxide (H_2_O_2_)^28^. After filtering the microplastics with GF/C filter, they were dried at 70 °C for 12 h in a hot air oven to completely dry, as preparation for further identification. Microplastics attached to the GF/C filter were photographed through a stereo microscope 40 × under ultraviolet light using UVA band (320 nm) with a high-resolution camera (3456 × 4608 pixels). The microplastic samples collected from the water and sediment produced 96 images (an average of 24 images/lap), whereas the benthic microplastics produced 150 images (an average of 33 images/lap, with the exact number varying depending on the monthly occurrence of benthic fauna). Furthermore, the microplastic samples attached to the GF/C filter were collected separately using tweezers by visual identification (shapes, colours and textures) into 10 groups following the standardised size and colour sorting (SCS) system^29^. These 10 groups were white fibre (FI-1), group of white twisted fibres (FI-2), black fibre (FI-3), blue fibre (FI-4), transparent fibre (FI-5), blue fragment (FR-1), transparent fragment (FR-2), turquoise fragment (FR-3), white fragment (FR-4), and orange fragment (FR-5). Next, ten samples from each group were randomly selected to be identified chemically. Fourier-transform infrared spectroscopy (FTIR) was used to determine the chemical compositions of the microplastic samples collected from wavelength range 4000 to 400 cm^−1^ in transmission mode using the Spotlight 200i model.

The results from the FTIR identified that FI-1 and FI-2 were Cotton Polyester Blend, FI-3, FI-4 and FI-5 were Polyester, FR-1 and FR-4 were Polypropylene, FR-2 was Low-density polyethylene FR-3 was Polyvinyl chloride and Polyethyl cyanoacrylate and the last group FR-5 was Alkyd. From these results the colours under UV light of particles in each group were also recorded. The results of the chemical and visual identification process for microplastics were adopted during the labelling step.

### Image labelling

**Figure 2:**
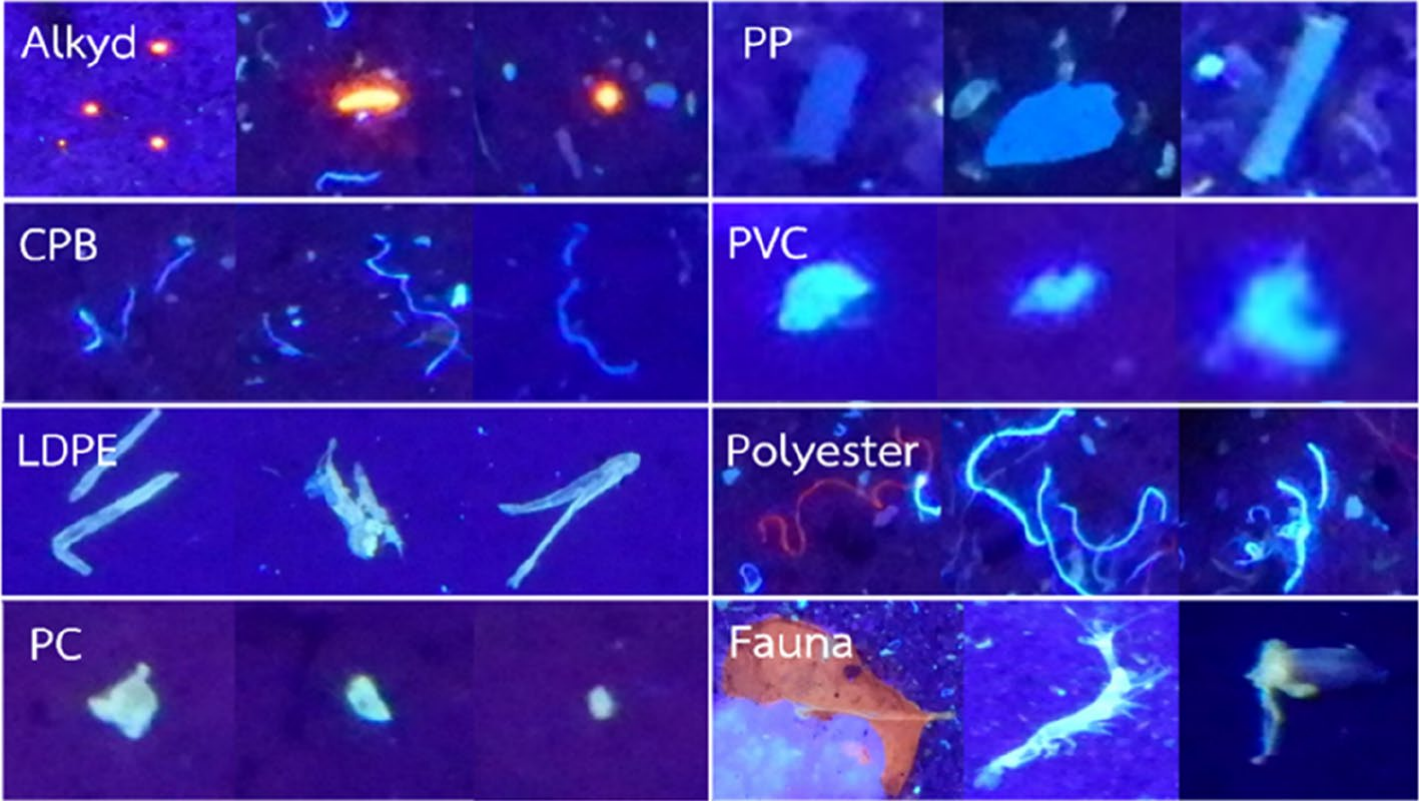
Examples of different microplastic types under UV light.

Object detection in computer vision required manual annotation using expert knowledge. This is a crucial step that can affect the classification model’s effectiveness. Each photograph was uploaded to COCO Annotator^30^, a web-based annotation tool, that was used to manually record the type of microplastic captured. Each piece of microplastics in the images was drawn in a tight bounding box. The results from FTIR and visual identification were applied as ground-truth to annotate the microplastic components through COCO Annotator. Under UV light, various microplastics reflect in different ways, so the fluorescent properties of microplastic can imply the type of polymer. FTIR was also adopted to verify the polymer type. The labels fell into 8 categories (Fig. 2): Fauna, which refers to non-plastics in photographs (benthic fauna, leaves, rocks, and broken glass), Alkyd (orange fluorescent pellets), CPB (Cotton Polyester Blend; blue fibres with low fluorescent), LDPE (Low-density polyethene; fluorescent blue), PC (Polyethyl cyanoacrylate; glows light green), PP (Polypropylene; sample blue but not fluorescent), PVC (Polyvinyl chloride; fluorescent blue) and Polyester (blue and some red fluorescent fibres).

After labelling all images in a tight bounding box from the real environments, there were 5720 objects spread across 230 images. The CPB had the highest number of microplastic objects which was 1844 instances, followed by Fauna (1435 objects) and Polyester (1293 objects). The top 3 lowest microplastics instances were LDPE, PC and PP which had 7, 10, and 136 objects, respectively. The remaining were Alkyd and PVC which had the counts 488 and 507, respectively. The dataset’s object instances were tiny, as the median size of all microplastic instances in this study was 39 × 39 pixels and the object area varied from 72 to 51909 pixels. The statistics of microplastic size (in pixels) of each class are shown in Table 1 including the number of objects in the original images (#original), the min area, and the area at 25, 50 and 95 percentiles (the max area was excluded due to an error when transforming data from the annotation platform). Using only this original data for training may not be enough. In order to balance the objects by class and help increase the quantity of images and objects, data augmentation and data creation were adopted.

**Table 1:** Summary statistics of microplastic size and number by polymer type collected from the study areas.

Polymer type	Min area	Area @ 25%	Area @ 50%	Area @ 95%	#Original*	#Generated**
Alkyd	132	667	1184	3212.8	488	2177
CPB	171	1174.5	2024	10,757.9	1844	2419
Fauna	108	834	1386	29,623.6	1435	2639
LDPE	34,124	67,043.5	89,000	123,835.9	7	2224
PC	360	484.5	739	4492.9	10	2146
PP	72	211.5	374	1843.05	136	2222
PVC	99	272	399	1452.6	507	2266
Polyester	216	1178	2040	12,097.9	1293	2419
Overall	72	837	1520	11,813.2	5720	18,512

*The number of objects in the original images.

**The number of objects in generated images performed in the data creation process.

### Image pre-processing and dataset creation

To improve the details in the images and minimize noise and uninteresting areas, image processing was used. The processes in this step were developed using Python language with OpenCV library (https://opencv.org/). First, a dark background was applied to exclude noise and other elements that were not in the designated area of main interest. This stage involved detecting the microplastics that were attached to the GF/C filter in the inner circle area; the outer part of the circle area was mask out. Second, the images were added cold colour and the saturation increased to enhance the colour and details of the object. Microplastic classification using deep learning required a large number of labelled data for training a model. However, the number of microplastics in some classes was low. So, image creation was required. Moreover, the microplastic images were imbalanced in each category, which interferes with the model learning^31^. This motivated the creation of new images to ensure that the number of microplastics in each category is balanced and large enough for training. To generate a new image, the idea from Copy-Paste data augmentation method^32^ was applied. First, several background photos that contain fewer microplastic objects were selected. Second, microplastic objects were randomly selected and overlaid with balanced distribution by class, and still keeping the number of objects in each image unchanged from the original. Next, each object was rotated and flipped by a random angle (0°, 90°, 180°, or 270° to match the pixel pattern), and randomly resized (to 0.6, 0.8, 1, 1.5, 2, 2.5 or 3 fold linear expansion). Lastly, the generated images were saved, and the dataset was then split into training, testing, and validation sets in the proportions 80:10:10. The original images also separated into training, testing, and validation set the same ratio as the generated images. Additionally, an external testing set was adopted from different study area but with comparable environmental control, which performed the same pre-processing and labelling process.

**Figure 3:**
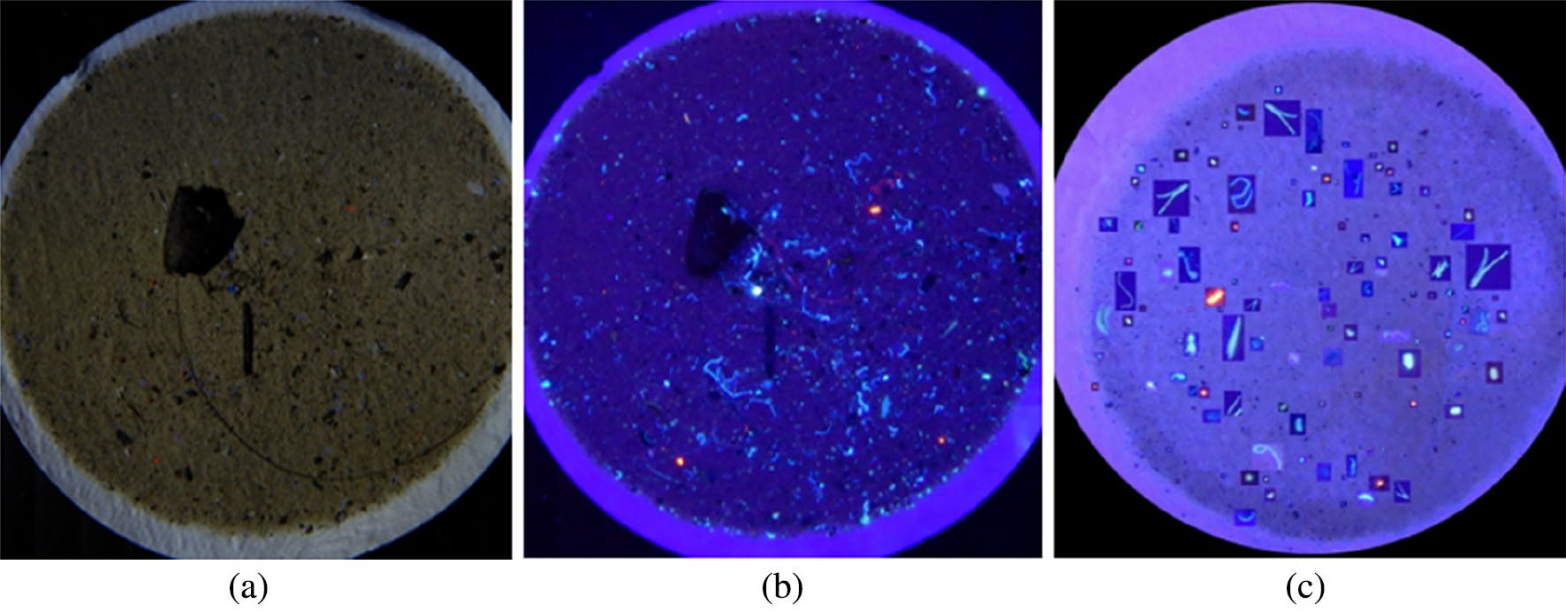
An example of microplastic images. (**a**) A microplastics image under natural light, (**b**) same microplastics image under UV light, and (**c**) a generated microplastics image.

Adding a black background to an uninteresting region aided in the removal of noise and other objects that are not relevant to the subject of interest. Adding cold colour and increasing saturation, on the other hand, did not improve training, since a photo taken under UV light was sufficient to enhance microplastic details. The microplastics photo dataset was generated from the original 230 images to 650 images for the training set and 65 images for the testing set. Figure 3 shows examples of microplastics images, including a microplastics image under natural light (Fig. 3a), the same microplastics imaged under UV light (Fig. 3b), and a generated image (Fig. 3c). The results of the increase in the number of generated images are shown in the last column (#generated) in Table 1 which has a total number of objects from 5720 instances, increasing the number of objects to 18,512 instances.

### Microplastics classification

Classifying and counting the number of microplastics is a problem of object detection and instance of semantic segmentation. Due to pixel-wise labelling, segmentation algorithms are time-consuming, and compute and memory expensive. Even though pixel-wise approach might produce good results, the object detection method was used in this study. Object detection can be divided into two types: two-stage object detectors (e.g., RCNN, Fast-RCNN, Faster-RCNN, Mask R-CNN, etc.) and one-stage object detectors (e.g., YOLO, SSD, RetinaNet, etc.)^33^. One-stage detectors are normally significantly faster than the two-stage detectors, although they produce less accurate results. The Faster R-CNN model with a ResNet-50-FPN backbone^34^ was selected due to its high performance in detecting small objects according to Liu et al. Python language and PyTorch library (https://pytorch.org/) was used to implemented the model. The Faster R-CNN is composed of 3 parts: (1) convolutional neural network (CNN) to extract the appropriate features and classify image region, (2) region proposal network (RPN) to predict the bounding box of the objects, (3) region-based convolutional neural network (R-CNN) to predict object class for all bounding boxes. The 50-layer Residual Neural Network (ResNet-50) was adopted as a backbone model in the feature extraction process (first part of Faster R-CNN). Transfer learning was also applied, which helps reduce the training time and allows a comparatively small training dataset. As the size of the objects was varied, Feature Pyramid Network (FPN) can assist in better detection. When the Faster R-CNN model with a ResNet-50-FPN backbone was ready in Python code, the labelled images from the pre-processing process were used as input data for training the microplastic models which needed to find-tune the parameters of the models for the best result. The highest accuracy model was used for classifying the type of microplastic.

### Evaluation metrics

The microplastic classification performance was assessed using the following evaluation methods that characterize the model's accuracy and validity.

#### Confusion matrix

A confusion matrix was used to evaluate the classification performance of microplastics. The classification results were obtained from the actual dataset and the prediction dataset^35^, and the matrices had each the size of n × n, where n denotes the number of microplastic species. If n is 2 for microplastic and non-microplastic classes, a true positive (TP) outcome occurs when the model correctly predicts the microplastic class, a true negative (TN) outcome occurs when the model correctly predicts the non-microplastic class, a false positive (FP) outcome occurs when the model incorrectly predicts the microplastic class, and a false negative (FN) outcome occurs when the model incorrectly predicts the non-microplastic class. The following criteria were used to assess the classification efficiency of microplastics: precision, recall, and F1 score, which can be calculated using the Eqs. (1)–(3). The proportion of accurately detected microplastics among all of the candidate microplastics is known as precision. Recall is the proportion of actual microplastics that was successfully identified. The F1 score combines recall and precision.

1 $$precision = \frac{TP}{{TP + FP}}$$

2 $$recall = \frac{TP}{{TP + FN}}$$

3 $$F1 = \frac{2 \times precision \times recall}{{precision + recall}}$$

If the suspected object was detected by the model, the bounding box (bbox) object’s confidence score is checked to see if it is greater than or equal to the specified threshold. (This study required greater than 0.4 level of confidence.) Accept and display the bbox of the selected object in the image. However, to determine which bbox is TP, Intersection over Union (IoU) was used to evaluate the overlap of the ground truth region and prediction region. IoU values are between 0 and 1 where 0 denotes an absence of overlap and 1 denotes complete overlap. The threshold of this IoU can both decrease the loss of tiny object data during training and improve small object detection accuracy^33^. In this study, the IoU threshold was set at 0.5, a standard of PASCAL VOC’s measure^36^, and employed to determine which bbox is TP and which is FP. The bbox is TP if the IoU score is larger than 0.5 and FP otherwise.

#### Precision–recall curve

**Figure 4:**
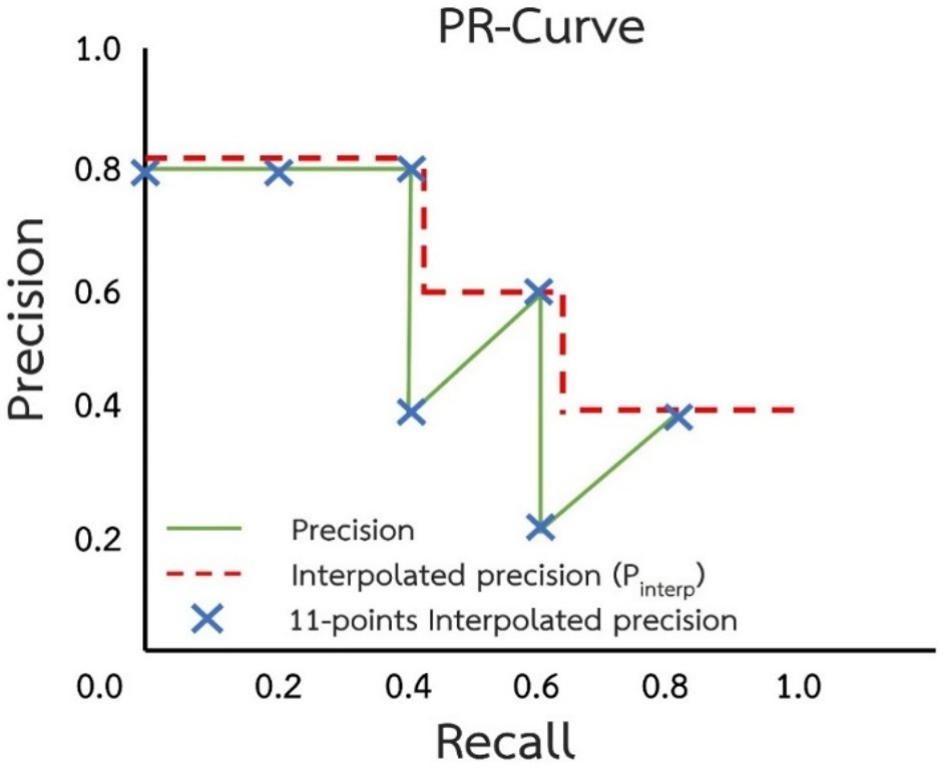
Example of precision recall curve.

After determining the precision and recall of data, these were plotted in the same graph for the precision–recall curve (PR-Curve) as shown in Fig. 4, and the area under the curve was determined^37^. If the frame overlay is not microplastic, the precision is reduced, and the recall is constant. Overlays of microplastics increase precision and recall values and shift them to the right. After drawing, the graph was restyled to make it easier to read. Interpolated precision was obtained by omitting serrated lines and drawing a line from the maximum precision (*p*_*interp*_).

#### Mean average precision (mAP)

Mean average precision is the average precision (AP) of all microplastics averaged^38^. The AP values are calculated from the area under the precision curve and recalled from the PR-Curve. The equation for this calculation is as follows:

4 $$AP = \mathop \sum \limits_{i = 1}^{n - 1} (r_{i + 1} - r_{i} )p_{interp} (r_{i + 1} )$$

where r denotes the recall value for each level at which the precision changes for the first time, n denotes the total number of images, and *p*_*interp*_ denotes the new precision value, with the maximum value equal to the recall value at each level.

5 $$mAP = \mathop \sum \limits_{i = 1}^{K} \frac{{AP_{i} }}{K}$$

in which the total number of classes is denoted by K, and the mean accuracy of the i-class is denoted by $AP_{i}$.

## Results

In Python 3.7, the model was implemented using the PyTorch library (https://pytorch.org/). The microplastic classification model had the highest accuracy when the following parameters were configured. The stochastic gradient descent (SGD) optimizer was used to minimize the loss on training the model, with a learning rate of 0.03, a momentum of 0.9, and weight decay of 0.0001. The model was trained on a GeForce RTX 2080Ti GPU. The model was unable to learn when trained directly from the original dataset, and the mAP was extremely low due to the small number of images and imbalanced objects in each class. Then, the training process was separated into two steps. The first step was training from generated images using COCO train2017 as pre-trained weights in the Faster R-CNN model. The model was trained with 45 epochs, and the learning rate scheduler which decreases the learning rate by 10 × every 25 epochs was used. Because the created dataset has class balance, this initial phase was employed to decrease the problems of class imbalance from the original dataset. Furthermore, the created dataset aided in increasing the number of training images, which improved the model’s accuracy and reduced the problem of overfitting. However, the generated dataset increased the possibility of identical objects from lower classes being duplicated, which resulted in poor prediction accuracy for those classes. The mAP for the validation and test sets of the generated images were 0.877 and 0.927, respectively. The second step was training from original images in the training set using the pre-trained model from the first step. The learning rate scheduler, which reduces the learning rate by 10 × per 8 epochs, was used to train the model across 20 epochs. This step included retraining the model using the original dataset, which aids in fine-tuning the model’s ability to predict labels for items in the actual photos. The mAP for the validation and test sets, and an external test set of the original images were 0.382, 0.339 and 0.357, respectively. Moreover, the area under the PR-curve revealed that Alkyd plastics had the highest average precision (AP) at 0.67, followed by LDPE (0.50), Polyester (0.48), CPB (0.46), Fauna (0.46), PVC (0.24), PP (0.12), and the least was for PC (0.00).

**Figure 5:**
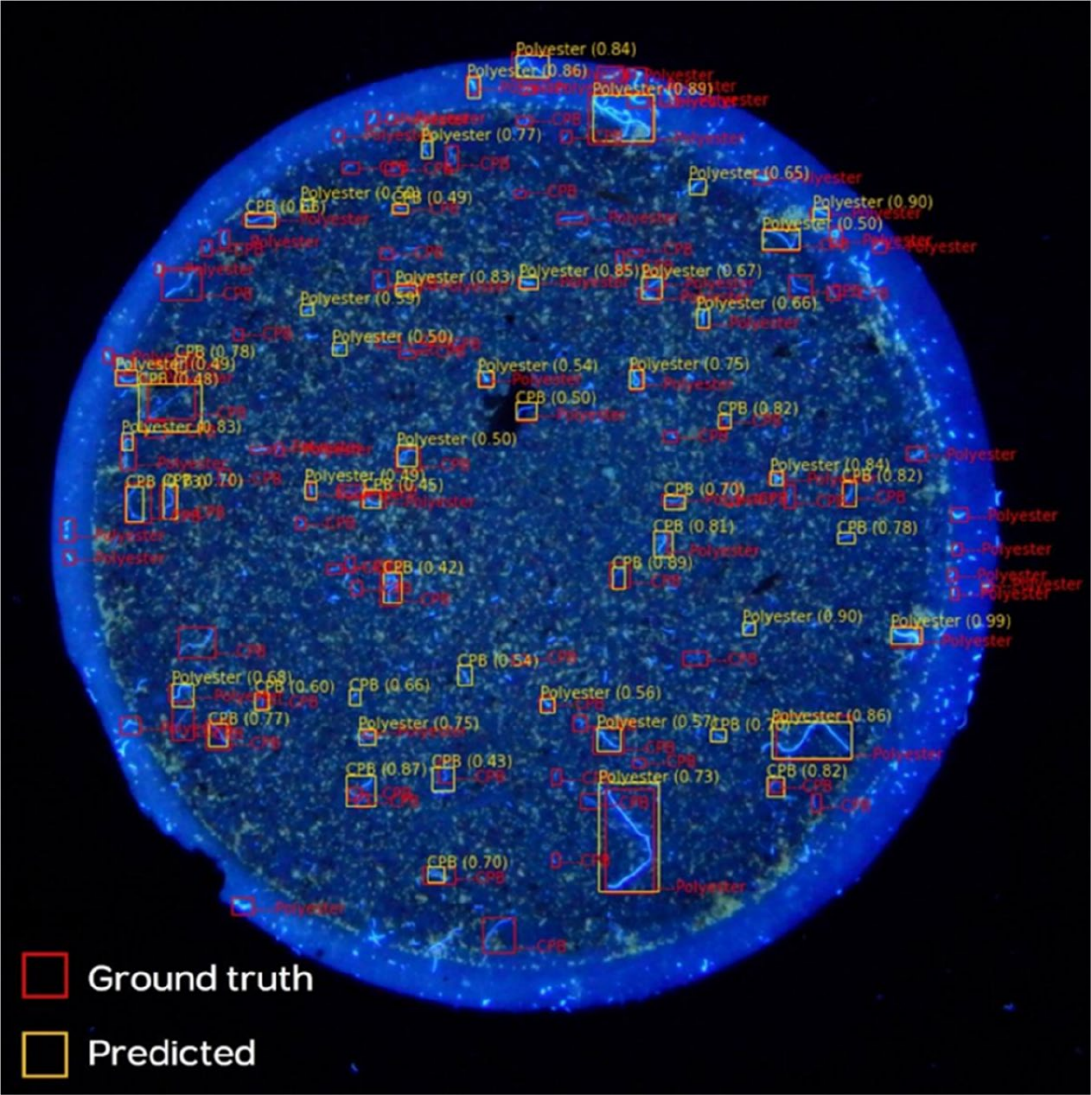
An example of microplastics image taken through a microscope under UV light with ground truth and predicted bounding boxes.

The predicted object is presented in a bbox with the label and confidence score of each class above the bbox in Fig. 5. The confusion matrix (CM) in Table 2 quantifies the classification efficiency of microplastics in the test dataset of the original images. In Table 3 the last row (obj_gt) shows the number of labelled objects in each class (ground truth), whereas the row of None shows the number of predicted but unlabelled items. We, however, left the ‘None’ scenario first because we were unable to label all the microplastics present in the photos. We additionally removed LDPE, PC, and PP in this scenario, since the original training data for these three classes was too limited (Table 1). So, based on this scenario the overall precision, recall and F1 scores were 0.878, 0.361, and 0.494, respectively, and the details by each class are shown in Table 3. The Alkyd got the highest F1 score (0.786) followed by Polyester (0.578), CPB (0.457), Fauna (0.377) and PVC (0.273), respectively. After inspecting all the predicted items in the instance of None, we discovered that practically all of the predicted things that were not labelled were correct. As a result, we recalculated the precision, recall, and F1 score based on the assumption that the prediction in the ‘None’ scenario is 80% accurate (Table 3). Precision, recall, and F1 score had all been improved to 0.861, 0.455, and 0.585, respectively. Alkyd received the highest F1 score (0.787), followed by Polyester (0.629), CPB (0.587), Fauna (0.559), and PVC (0.365).

**Table 2:** The confusion matrix for the internal test set of the original images. Significant values are in bold.

Polymer type		Predicted							
		Alkyd	CPB	Fauna	LDPE	PC	PP	PVC	Polyester
Actual	Alkyd	**11**	0	0	0	0	0	0	0
	CPB	0	**69**	1	0	0	0	0	9
	Fauna	0	1	**30**	0	0	0	0	1
	LDPE	0	0	0	**1**	0	0	0	0
	PC	0	0	3	0	**0**	0	0	0
	PP	0	0	1	0	0	**0**	0	0
	PVC	0	4	0	0	0	0	**9**	0
	Polyester	0	14	2	0	0	0	1	**85**
	None	1	92	60	0	0	5	7	44
	obj_gt	17	214	122	2	5	38	56	199

**Table 3:** Accuracy in the internal and external test set of the original images excluding and including ‘None’ scenario with 80% correct.

Polymer type	Internal test set Exclude the ‘None’ scenario			Internal test set Include the ‘None’ scenario with 80% correct			External test set Excluding the ‘None’ scenario		
	Precision	Recall	F1	Precision	Recall	F1	Precision	Recall	F1
Alkyd	1	0.647059	0.785714	0.983333	0.655556	0.786667	–	–	–
CPB	0.784091	0.32243	0.456954	0.792222	0.466013	0.586831	0.783217	0.260263	0.390698
Fauna	0.810811	0.245902	0.377358	0.804124	0.428571	0.55914	0.930845	0.450345	0.607015
PVC	0.9	0.160714	0.272727	0.858824	0.231746	0.365	–	–	–
Polyester	0.894737	0.427136	0.578231	0.864748	0.49465	0.629319	0.850972	0.242611	0.377575
Overall	0.877928	0.360648	0.494197	0.86065	0.455307	0.585391	0.855011	0.31774	0.458429

We also verified the model’s accuracy with an external test set of microplastic photos from a different research location but with comparable settings (120 images). This external test set, on the other hand, had mostly CPB (1291 objects), Fauna (1883 objects), and Polyester (1624 objects) categories. The total precision, recall, and F1 score with ‘None’ excluded scenario were 0.855, 0.318, and 0.458 respectively (Table 3). It can be seen that the precision and recall of both the internal and the external test sets were comparable, confirming that the model can predict the same behaviour with photographs under UV light using a high-resolution camera. Additionally, our microplastic classification model’s prediction time from a single microplastic image with contain 21 objects in the median (or 1 to 444 objects per image) was around 4 seconds.

## Discussion

To the best of our knowledge, this is the first study using deep learning to classify microplastic types from images of microplastics collected in the field and photographed through stereo microscope under UV light. As a result, direct comparison with other studies may be challenging. Therefore, the following subsections will cover: classifying the type of microplastic under UV light; machine learning methods for detecting and classifying microplastics; deep learning model architectures for discriminating microplastic types; and comparing the accuracy of the same methods and architectures for small object detection.

We attempted to differentiate between several types of microplastics in our study, including Fauna, Alkyd, CPB, LDPE, PC, PP, and Polyester. Since the microplastic morphology is complicated, it is challenging to distinguish the type of microplastic manually under sunlight using only colour, shape, and size. So, we adopted UV light to enhance the ability to discriminate the microplastics. Different polymer types react to UV light in distinctive ways, resulting in different fluorescent colours^12^. Due to this advantage of UV light, we prepared and annotated microplastic images that were captured under UV light for use in training the deep learning model. The trained model, Faster-RCNN, from our study can automatically recognize many tiny plastic pieces and categorize the different types of polymers from an image at once. Moreover, our classification model was trained using a large number of thousands of microplastic pieces (4000–5000 objects from original dataset). A high-resolution camera was used to collect as much information as possible, and image resizing was restricted to prevent losing microplastic features. In contrast, the Meyers et al. study^12^ utilises reflectance values from images in Red Green Blue (RGB) data and applies a decision tree to distinguish between microplastics by focusing solely on colour and ignoring shape and size. Additionally, their work is slower and less effective than ours since their process must isolate each plastic particle into a single image for training and it can only label one particle from an image at a time. Furthermore, there were only about 200 plastic particles used in their study, which is a very small quantity.

Object detection and semantic segmentation are two techniques used in computer vision and image processing to discriminate and annotate objects into various categories. Object detection is the process of identifying each distinct object in an image and annotating the presence of microplastics within the bounding box. Semantic segmentation is another technique that can count and detect objects by labelling boundaries at the pixel level. Since segmentation approaches are time-consuming, requiring expensive computation and memory, our study used object detection instead of pixel-by-pixel labelling. However, Lorenzo-Navarro et al. combine semantic segmentation (U-Net) with image classification to separate particles from the surrounding environment and classify microplastics into fragments, pellets, and lines by their morphology^19^. Their study achieved very high precision and recall in classifying microplastics. The Lorenzo-Navarro et al. study reported a precision of 98.17% and a recall of 98.11%, while the Faster-RCNN model used in our study achieved lower precision and recall. However, it is much simpler to distinguish between different morphologies of microplastics than the actual polymer types as done in our study, because fragments, pellets, and lines are only considered in terms of shape, whereas only shape cannot classify into type of microplastics. In addition, it should be highlighted that the microplastics utilized in the research by Lorenzo-Navarro, et al. were fabricated and particularly created for laboratory use^19,39^, which differs from our study that used real samples from the lagoon.

Several model architectures have been utilized to classify objects, when it comes to object detection. Faster R-CNN model with a ResNet-50-FPN backbone was deployed in our study due to its high performance in detecting small objects^33^. Microplastic particles are similarly small in size to the Liu et al. study. However, Mask-RCNN is one of the most efficient models for identifying objects in bounding boxes. In the Wegmayr et al. study, which used the Mark R-CNN model to discriminate microplastic fibre type (single and tangled fibres), their precision was in the range 30–64% and recall in the range 32–63%^20^. The Faster-RCNN model in our study had a higher detection efficiency for polymer type, with precision and recall in the ranges 79.2–98.3% and 23.1–65.6%, respectively.

Since the microplastic particles are small, of size comparable with the Liu et al. study, we selected deep learning methods for the small object detection^33^. In their study the small objects are less than 50 × 50 pixels in images, which is almost similar size to ours. Object detection using Faster R-CNN in Liu et al. study achieved 35% from the DOTA dataset, 24.1% from COCO and SUN datasets and 33.6% from Wider Face dataset as mAP, and we can use these results as a baseline to assess our results. In comparison to Liu et al., our study's mAP scores were 33.9 % on the internal test set and 35.7 % on the external test set, which are almost similar. This suggests that the microplastic identification technique used in our study is in the same league as in other studies that classify tiny objects. As a result, the deep learning approach combined with microplastic imaging under UV light can reasonably well distinguish polymer types. However, because of the low recall, our method's quantification has to be improved.

To achieve better results, images should be taken at a higher resolution to avoid losing microplastic particle details^19^ and to avoid problems with imbalanced data, there should be enough photos for model training and testing^40^. Moreover, collecting more data to gather a diversity of microplastic samples in the real world is needed. Additional microplastic samples need to be collected and labelled, and a new model must be trained in order to improve the classification accuracy of LDPE, PC, and PP as well as to distinguish other polymer types. The semantic segmentation approach can improve the performance of the classification model. In summary, microplastic classification utilizing deep learning raises the current microplastic monitoring to a higher level and establishes an information technology application standard in the classification of microplastics.

## Conclusions

The approach of using deep learning with microplastic images taken through a microscope under UV light, as presented in this work, is valid and promising. This procedure also included data creation to increase the number of training images in a class-balanced manner. The accuracy of classification was high and met the standard of small object detection. Moreover, this approach reduced the microplastic discrimination and counting time. Also, this study is the first one to differentiate specific polymer types using deep learning from microplastic imagery under UV light.

## Data Availability

The paper includes all the information required to assess its conclusions. Additional information related to this paper can be requested from the Corresponding Author (Ponlachart Chotikarn).
